# Willingness to accept new TB vaccines among adults, adolescents and their caregivers in a high TB burden setting

**DOI:** 10.5588/ijtldopen.25.0177

**Published:** 2025-07-09

**Authors:** K.N. Nelson, L.M Cranmer, L. Vasudevan, A. Lima, S. Acacio, A. García-Basteiro

**Affiliations:** ^1^ Emory University Rollins School of Public Health, Department of Epidemiology, Atlanta, GA, USA;; ^2^ Emory University School of Medicine, Atlanta, GA, USA;; ^3^ Emory University Rollins School of Public Health Hubert Department of Global Health, Atlanta, GA, USA;; ^4^ Centro de Investigação em Saúde da Manhiça (CISM), Manhiça, Mozambique;; ^5^ Barcelona Institute for Global Health, ISGlobal, Barcelona, Spain;; ^6^ Centro de Investigación Biomédica en Red de Enfermedades Infecciosas (CIBERINFEC), Barcelona, Spain.

**Keywords:** tuberculosis, Mozambique, vaccine hesitancy, BCG

Dear Editor,

An effective TB vaccine for adolescents and adults would cumulatively avert 37.2–76.0 million cases and 4.6–8.5 million deaths before 2050.^1^ Over the next five years, several pivotal efficacy trials of vaccine candidates among adolescents and adults will report results,^2,3^ potentially leading to licensing one or more vaccines. Candidates include both novel vaccine products^4^ and re-vaccination with the Bacille Calmette-Guérin (BCG) and other whole-cell vaccines to ‘boost’ immunity in older age groups.^5^ Understanding potential barriers to uptake of TB vaccines is key to maximizing their population-level impact. Vaccine hesitancy is a key concern for adoption of other vaccines for adults and adolescents, as seen most recently for COVID-19 and HPV.^5^ Hesitancy may be driven by concerns about safety and low perceived benefits, and vary by age, gender, or previous experience with TB (i.e., knowing someone with TB or being previously diagnosed themselves).^6^ Characterizing attitudes about new TB vaccines can facilitate planning for vaccine introduction.

Mozambique has a high TB and TB-HIV dual burden, with an estimated TB incidence of 361 per 100,000 and approximately 25% of people living with HIV.^7,8^ This study, conducted from February to July 2024, involved a cross-sectional survey, which was based on the Behavioral and Social Drivers of Vaccination framework,^9^ among adults and adolescent-caregiver pairs in Manhiça district, southern Mozambique. Participants were recruited from two settings: households in the community and an outpatient TB clinic. The demographic surveillance system facilitated identification of households, which were visited by the study team to offer participation to all household members. Patients attending the clinic were approached as they left the consultation room. Eligible adolescents were between 9–17 years of age and eligible adults were over 17 years of age. Written consent was obtained from all participants >17. Written assent from participants and written parental consent was obtained for participants ≤17. We administered surveys to collect demographic information, participants' prior vaccination experiences, sources of information and beliefs about and their willingness to receive a novel tuberculosis vaccine or BCG booster assuming the vaccine was proven to be safe and effective (‘If it were available, would you be willing to receive a BCG booster/a new tuberculosis vaccine?’). This study was approved by ethical and scientific committees at Centro de Investigação em Saúde da Manhiça (CISM), Manhiça, Mozambique (CIBS-CISM/050/2023 and CCI/036/AGO/2023) and Emory University (study #00006480).

We enrolled 151 adults (median age 34, 61% female), 41 adolescents (median age 15, 66% female), and 49 caregivers (median age 26, 90% female). The adolescents of 9 caregivers were unable to be contacted and enrolled. Most respondents (n = 163, 68%) were female. About half of of participants (n=134, 56%) had been previously diagnosed with TB or had a family or friend diagnosed with TB. Among adults and adolescents, willingness to receive a new vaccine product was 77% (148/192) overall and to receive a BCG booster was 84% (162/192) overall (see Figure). There was a similar proportion of affirmative (‘yes’) responses among adolescents and adults (p = 0.10 for a novel product and p = 0.36 for a BCG booster). There was more uncertainty (‘maybe’ responses) about a novel vaccine product (n=29, 15%) than for a booster dose of BCG (n=19, 10%), though this was not statistically significant (p = 0.16). More participants with a previous TB diagnosis (themselves or a close friend or family member) were willing to receive a novel vaccine product (81%, 72/89), compared to participants without (74%, 76/103), although this was not statistically significant (p = 0.03).

**Figure.**
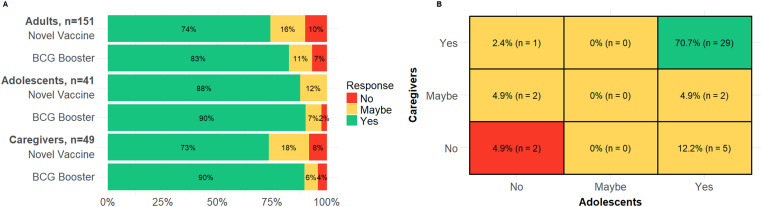
Willingness to accept a novel vaccine or a BCG booster among adults, adolescents, and their caregivers in southern Mozambique, 2024. **A**: Willingness to accept a novel vaccine or a BCG booster among adults, adolescents, and their caregivers, n = 241. **B**: Concordance in responses about willingness to accept a novel vaccine among adolescent-caregiver pairs (n = 41); green = both in pair responded ‘yes’; yellow = at least one respondent in pair responded ‘no’; red = both in pair responded ‘no’.

Among adolescents, willingness to receive a novel vaccine product (i.e., ‘yes’ responses) tended to be higher among girls (n=25, 93%) than boys (n=11, 79%) but was not statistically significant (p = 0.4); among adults, willingness was similar by gender (n=68, 74% among women and n=44, 75% among men, p = 1). Among adults, women expressed more uncertainty (n=20, 22%, responding ‘maybe’) than men (n=4, 7%) about a novel vaccine product (p = 0.03). Caregivers of adolescents were less likely to allow their child to receive a new vaccine (73%) than a BCG booster (90%), though this was not statistically significant (p = 0.07). Among adolescent and caregiver pairs, 76% (n = 31 out of 41 pairs) were concordant in their responses (i.e., Yes-Yes, Maybe-Maybe, or No-No). Of these, 94% (n = 29) both responded affirmatively (Yes-Yes).

Our survey indicates that there is a high overall willingness to receive a novel TB vaccine product or a BCG booster among adults and adolescents (the main target age group of vaccine candidates in the development pipeline), in southern Mozambique. However, the proportion willing to be vaccinated with a new TB vaccine (77%) is lower than reported coverage of neonatal BCG in Mozambique (93% in 2023)^10^ and willingness to receive other new vaccines indicated for adults. A recent meta-analysis estimated willingness to receive new malaria vaccines at >95% in malaria-endemic countries.^11^ Notably, our estimate of willingness to receive new vaccines is higher than some estimates of uptake of TB preventive therapy (TPT); for example, 50% in South Africa among eligible individuals.^12,13^

There were differences in willingness by vaccine product, age and gender. There was slightly higher willingness to receive a booster of a familiar vaccine (BCG) than a new product, suggesting some concern related to the ‘newness’ of a product rather than generalized concerns about vaccination. To the extent that ‘maybe’ responses indicate openness to but uncertainty about vaccination, it will be important to consider women as a target of vaccine outreach efforts. However, given the greater TB burden among men, acceptance in this group will be particularly important. Men were a minority (38%) in our study but future efforts to characterize attitudes towards new TB vaccines should aim for parity in recruitment. Differences in willingness to vaccinate by age and gender can be incorporated into mathematical models that aim to estimate vaccine impact. Recently published work has assumed coverage of new vaccines will reach 80%,^14^ which is within range of our estimates. However, coverage differences between population subgroups with varying burdens of TB (e.g., men and women, adolescents and adults) will be important for understanding differential vaccine impact in those groups.

Our study is the first to assess willingness to receive TB vaccines among the target group of the vaccine candidates most advanced in the pipeline (i.e., adults and adolescents), and to employ a paired design to assess concordance in vaccination decision-making of adolescents and their caregivers. The primary study limitations is the small convenience sample, which, given the context-specific nature of vaccine hesitancy, precludes statistically robust conclusions and limits generalizability of findings beyond southern Mozambique. Although our study population (median age of 25, IQR:18-44) falls squarely within the target group for vaccines (12–50 years^15^) with a similar proportion of adolescents as the general population (21% in our study, 24% in Mozambique in 2024), we intentionally oversampled people with previous TB (15% in our study) to understand vaccine perceptions in this key group at high risk for TB.
